# Ligand Binding Reveals a Role for Heme in Translationally-Controlled Tumor Protein Dimerization

**DOI:** 10.1371/journal.pone.0112823

**Published:** 2014-11-14

**Authors:** Andrew T. Lucas, Xiangping Fu, JingJing Liu, Mary K. Brannon, Jianhua Yang, Daniel G. S. Capelluto, Carla V. Finkielstein

**Affiliations:** 1 Integrated Cellular Responses Laboratory, Virginia Bioinformatics Institute, Department of Biological Sciences, Virginia Tech, Blacksburg, Virginia, United States of America; 2 Protein Signaling Domains Laboratory, Virginia Bioinformatics Institute, Department of Biological Sciences, Virginia Tech, Blacksburg, Virginia, United States of America; University of South Florida College of Medicine, United States of America

## Abstract

The *t*ranslationally-*c*ontrolled *t*umor *p*rotein (TCTP) is a highly conserved, ubiquitously expressed, abundant protein that is broadly distributed among eukaryotes. Its biological function spans numerous cellular processes ranging from regulation of the cell cycle and microtubule stabilization to cell growth, transformation, and death processes. In this work, we propose a new function for TCTP as a “buffer protein” controlling cellular homeostasis. We demonstrate that binding of hemin to TCTP is mediated by a conserved His-containing motif (His^76^His^77^) followed by dimerization, an event that involves ligand-mediated conformational changes and that is necessary to trigger TCTP's cytokine-like activity. Mutation in both His residues to Ala prevents hemin from binding and abrogates oligomerization, suggesting that the ligand site localizes at the interface of the oligomer. Unlike heme, binding of Ca^2+^ ligand to TCTP does not alter its monomeric state; although, Ca^2+^ is able to destabilize an existing TCTP dimer created by hemin addition. In agreement with TCTP's proposed buffer function, ligand binding occurs at high concentration, allowing the “buffer” condition to be dissociated from TCTP's role as a component of signal transduction mechanisms.

## Introduction

The translationally-controlled tumor protein (TCTP) is a highly conserved and ubiquitously expressed eukaryotic protein whose cellular function spans from mechanisms of cell growth and division to cytoskeleton reorganization and cell morphology (for review see [1]). TCTP expression is highly regulated and responds to numerous extracellular signals and intracellular conditions. For example, TCTP levels vary considerably in response to tissue specific growth factors, cytokines, and stress signals including those triggered by heat shock, starvation, pro-apoptotic conditions, the presence of environmental pollutants and heavy metals, and by changes in cellular calcium (Ca^2+^)-mediated homeostasis [2]–[6].

Despite its broad regulation and abundance, knowledge of TCTP function has remained elusive. The best classification of TCTP's role in cellular function places the protein into two groups: *i*) functions associated with cell growth, division, and death and *ii*) immunity/allergic-related functions. Initially, evidence of a role for TCTP in cell death arose from variations in the cell's phenotype under conditions in which the protein was either overexpressed or its gene was knocked down, resulting in enhancement of the action of anti-apoptotic players or in prevention of pro-apoptotic components from triggering cell death, respectively [7]–[10]. Other evidence establishes a role for TCTP in cell proliferation. This includes *i*) the regulation of the GTPase activity of the *Drosophila* Ras homologue Rheb, a direct target of TSC1/2 tumor suppressors responsible for tuberous sclerosis [8], *ii*) the stabilization of the GDP form of the translational elongation factor eEF1A [11], and *iii*) progression through cytokinesis by a mechanism that involves phosphorylation of TCTP by the polo-like kinase and, consequently, reduction of the microtubule-stabilizing activity of TCTP [12],[13]. In agreement with a role for TCTP in cell growth and proliferation, this protein is overexpressed in tumor cells and its inhibition by antisense or siRNA promotes apoptosis or, in other cases, induces the reorganization of cells into specific structures when the malignant phenotype has been suppressed (*i.e.*, in MCF-7 and T47D breast cancer cells) [14]. In addition, TCTP exhibits extracellular function by acting as an IgE-dependent histamine-releasing factor [HRF; [15]]. As shown, fluids secreted from human lung macrophages were able to induce Ca^2+^-dependent HRF release from basophiles and mast cells in an IgE-dependent manner [16]–[19]. Unlike its mononuclear cellular activated version, the serum form of human HRF (similar to extracellular TCTP) exhibits cytokine-like activity *in vivo* when dimerized, an event that is independent of post-translational modifications and thought to be mediated by a largely unknown player(s) [20].

The most well-characterized compound that binds TCTP is artemisinin, a natural sesquiterpene endoperoxide that is selectively toxic to malaria parasites [21]. Artemisinin's mode of action is simple in concept. When the malaria parasite *P. falciparum* infects erythrocytes, it digests most of the host-hemoglobin (∼80%) for its vital needs releasing high quantities (in the millimolar range) of free heme (Fe^2+^-protophorphyrin IX). Because heme is toxic to the parasite and cannot be secreted, heme is converted into an insoluble crystalline form called hemozoin that acts as a detoxification agent and accumulates in the digestive vacuole of *Plasmodium falciparum*-infected erythrocytes [22]. Artemisinin interferes with the production of hemozoin by reacting with heme, thereby, allowing maintenance of the toxic high heme environment, which kills the parasite [23]. Heme is speculated to mediate artemisinin's binding to TCTP, thus, interfering with TCTP's multifunctional cellular role and enhancing artemisinin's antimalarial activity [24].

Calcium is another ligand that plays a relevant role in TCTP biology by modulating TCTP expression both at the transcriptional and post-transcriptional levels while influencing its function through direct binding to a yet unidentified motif [25], [26]. Solution structure studies of TCTP using nuclear magnetic resonance (NMR) spectroscopy show that binding occurs within a noncanonical Ca^2+^-binding domain conserved among TCTP family members [27]. Although weak, Ca^2+^ binding to TCTP seems important for maintaining cell homeostasis and Ca^2+^ transport [28]. This is particularly relevant in a system where Ca^2+^ concentration varies greatly, typically from 10–100 nM, in the cytosol of eukaryotic cells to millimolar levels in both the extracellular environment and the lumen of the endoplasmic reticulum, the major Ca^2+^ storage compartment in the cell [29]. As a result, TCTP has been proposed to belong to a new class of Ca^2+^-binding proteins where the traditional EF-hand and CalB domains are largely absent.

In this work, we further explored TCTP ligand interactions and established the need for a conformational change associated with hemin binding to TCTP that mediates its dimerization and stability, an event that is reversed by Ca^2+^ binding. As a result, we propose that TCTP acts as a cellular “buffer” by impeding the toxic accumulation of free heme and sequestering Ca^2+^ under various physiological and pathological scenarios. Furthermore, TCTP association to either ligand influences its oligomeric state, suggesting the existence of a region within TCTP that responds to different cellular signals.

## Materials and Methods

### Plasmid constructs

The human *tpt1* cDNA was cloned into the *SalI* and *NotI* sites downstream of the glutathione S-transferase (GST) gene in the pGEX-4T-3 vector. For transfection experiments, *tpt1* cDNA was cloned into a pCS2+*myc*-tag vector modified for ligation-independent cloning (Novagen).

### Protein sequence alignment

Protein sequences of TCTP were obtained from the NCBI database and were aligned using CLUSTALW. Accession numbers are *Xenopus laevis* (Q7ZYF2), *Labeo rohita* (Q98SJ7), *Brachydanio rerio* (Q9DGK4), *Homo sapiens* (P13693), *Bos taurus* (Q862G3), *Sus scrofa* (P61288), *Oryctolagus cuniculus* (P43348), *Mus musculus* (P14701), *Rattus norvegicus* (Q6P9V3), *Gallus gallus* (P43347), *Drosophila yakuba* (Q6XIN1), *Bombyx mori* (Q75VN3), *Anopheles gambiae* (Q7QCK2), *Dermacentor variabilis* (Q8T9S3), *Caenarhabtitis elegans* (Q93573), and *Lumbricus rubellus* (018477). *A. gambiae* has an extra stretch of 14 residues (FLLVGQKFSPSSNK) that is not present in any TCTP from other species and was removed in order to facilitate sequence alignment.

### Purification of recombinant proteins and chromatography analyses

TCTP and its His^76^Ala-His^77^Ala mutant form, were prepared as N-terminus fusions with glutathione S-transferase. The chimeric protein was expressed in *E. coli Rosetta* strain (Novagen) and purified by glutathione-sepharose chromatography following manufacturer's instructions (GE Healthcare). Untagged proteins were obtained by digestion of fusion proteins with thrombin (1 U/20 µg of GST-TCTP) for 1 h at room temperature. Reactions were stopped by the addition of 5 mM dithiothreitol. Untagged proteins were resolved by fast performance liquid chromatography (AKTA UPC-900, GE Healthcare) using a 16/60 Superdex 75 column. Protein was loaded onto a 120-ml column equilibrated with 50 mM Tris-HCl, pH 7.8, 250 mM NaCl, and 1 mM EDTA. Peak fractions were pooled and concentrated to about 2 mg/ml in a Viva-Spin 5,000 MW cut-off device (GE Healthcare). When samples were analyzed for heme and Ca^2+^-mediated oligomerization, proteins were loaded onto a similar column equilibrated with either 50 mM Tris-HCl, pH 7.8, 250 mM NaCl, and 1 mM hemin [Fe(protoporphyrin IX)Cl] or 50 mM sodium citrate, pH 6.0, 250 mM NaCl, and 50 mM CaCl_2_, respectively.

### NMR analysis

NMR spectra were acquired at 35°C using a Bruker Avance 800 MHz spectrometer (University of Virginia). Binding of ligands to TCTP was investigated using 0.2 mM uniformly ^15^N-labeled TCTP in 50 mM sodium phosphate, pH 7.8, containing 200 mM NaCl, and 10% ^2^H_2_O and visualized in heteronuclear single quantum coherence (HSQC) spectra in the absence or presence of increasing amounts of hemin up to a final molar ratio of hemin:TCTP of 8∶1. In other experiments, TCTP was pre-incubated with a 1∶4 molar ratio of TCTP:hemin for 1 h at room temperature prior to addition of 5 mM CaCl_2_. After data collection, 10 mM EDTA was added to the same sample and the HSQC spectrum of the protein recorded. Spectra were processed with NMRPipe [30] and analyzed with NMRPipe and nmrDraw [31]. Sequence-specific resonance assignments of TCTP were obtained from [27].

### Non-denaturing gel electrophoresis analysis

Samples of untagged TCTP (8 µg) were pre-incubated with various concentrations of either Ca^2+^ (up to 1∶2,381 TCTP: Ca^2+^) or hemin (up to 1∶32 TCTP:hemin) for 1 h at room temperature before adding 50% glycerol. Electrophoresis was carried out in a 6% polyacrylamide gel of constant pH in Tris-glycine buffer (25 mM Tris, pH 9.0, 240 mM Glycine) at 4°C and at a constant voltage of 100 V for 70 min. Proteins were visualized by Coomassie blue staining.

### Chemical cross-linking

A cross-linking reaction of either TCTP or GST (positive control) was performed in the presence of the chemical crosslinker bissulfosuccinimidyl suberate (BS3). Proteins (5 µM), in the absence and presence of either hemin (32 µM) or CaCl_2_ (50 mM), were incubated with fresh BS3 (2.5 mM) in 100 mM HEPES (pH 7.5) for 1 h at room temperature. Reactions were stopped by the addition of 1 M Tris-HCl (pH 8.0) and samples were resolved by SDS-PAGE.

### Circular dichroism spectroscopy

Far-UV circular dichroism (CD) spectra were obtained on a Jasco-815 spectropolarimeter using a 1 mm slit-width cuvette. Untagged TCTP protein (5 µM in 50 mM Na citrate, pH 6.0, and 100 mM KF) was titrated against increasing concentrations of both hemin (from 1∶0.125 to 1∶32 TCTP:hemin molar ratio) in 5 mM Tris-HCl, pH 8, 100 mM KF and Ca^2+^ (from 1∶200 to 1∶10,000 TCTP:Ca^2+^ molar ratio) in 50 mM sodium citrate, pH 6.0, and 100 mM KF. Five accumulated scans for each sample were recorded from 190 to 260 nm with an increment of 0.5 nm, a scan rate of 50 nm min^−1^, a response time of 4 s, and a sensitivity of 50 mdeg at room temperature. All CD spectra were corrected by subtraction of the background from the spectrum obtained with either buffer alone or buffer containing either hemin or Ca^2+^. Raw data were converted to mean residue ellipticity, θ, in degrees cm^2^ dmol^−1^. Data were analyzed for protein secondary structure using DICHROWEB [32] and deconvoluted using CDSSTR [33].

### Urea denaturation assays

A Jasco-815 spectropolarimeter equipped with a thermoelectric temperature controller was used to obtain denaturation data using a 1 mm slit-width cuvette. TCTP (5 µM) was pre-incubated in the absence and presence of both hemin (1∶16 TCTP:hemin molar ratio in 5 mM Tris-HCl, pH 8, 100 mM KF) and Ca^2+^ (1∶10,000 TCTP:Ca^2+^ in 50 mM Na citrate, pH 6.0, and 100 mM KF) for 1 h at room temperature before urea addition. Titration was performed by equilibrating the samples with increasing concentrations of urea (250 µM to 8 M). Five accumulated scans for each sample were recorded from 190 to 260 nm with an increment of 0.5 nm, a scan rate of 50 nm min^−1^, a response time of 4 s, and a sensitivity of 50 mdeg at 30°C. Blanks were subtracted from the spectra. Data points at 222 nm were used to construct kinetic curves [34].

### Intrinsic fluorescence measurements

Steady-state fluorescence emission spectra were recorded in quartz cells at 25°C using a Jasco-815 spectropolarimeter equipped with a thermoelectric temperature controller. The excitation wavelength was set at 295 nm and the emission wavelength spectra were obtained from 310 to 410 nm; the integration time was 0.1 s and the slit-widths set at 5 nm. TCTP (1 µM) was prepared in the hemin and Ca^2+^ buffers described above. Titrations were performed by adding increasing amounts of either hemin or Ca^2+^ to TCTP at the indicated ligand concentration ranges. Background spectra (buffer blank, in the absence or presence of either Ca^2+^ or hemin) were collected under similar conditions and subtracted to obtain the final fluorescence spectra. Binding constants and best-fit traces were generated by fitting to a nonlinear regression equation using Kaleidagraph (Synergy Software, Reading, PA).

### Analytical ultracentrifugation

Experiments were performed at the Center for Analytical Ultracentrifugation of Macromolecular Assemblies (CAUMA) at the University of Texas Health Science Center, San Antonio (UTHSCSA) using a Beckman Optima XL-I analytical centrifuge with absorbance and interference optical detection systems (Beckman Coulter). Sedimentation velocities were analyzed using the UltraScan software suite as described [[35], http://www.ultrascan.uthscsa.edu] and calculations were performed at the Bioinformatics Core Facility at UTHSCSA. The TCTP sample (MW 19,604 Da) was prepared in a buffer containing 20 mM Tris-HCl, pH 8, and 150 mM NaCl. The TCTP:hemin complexes were prepared in the same buffer containing increasing amounts of hemin [0.5 to 15 µM, MW_hemin_: 651.94, Frontier Scientific). Absorbance data were simultaneously acquired at wavelengths of 230 and 280 nm, at 20°C, and at a rotor speed of 60,000 rpm (250,000xg) using standard double-channel centerpieces. The concentration of TCTP was about 0.4 µM (OD_230_ = 0.45) in all sedimentation experiments and its partial specific volume was 0.7337 cm^3^/g at 20°C. Data were first subjected to 2D spectrum analysis with simultaneous removal of time-invariant noise [36] followed by enhanced van Holde-Weischet analysis [37], genetic algorithm refinement [38], and Monte Carlo analysis [39].

### Limited trypsin proteolysis

Twenty microliter reaction mixtures containing TCTP (6 µg) and bovine trypsin (15 ng/µl) in 20 mM HEPES, pH 7.5, were incubated at room temperature for various times. Reactions were stopped by the addition of Laemmli sample buffer followed by 5 min of boiling. In other experiments, TCTP samples were pre-incubated for 1 h at room temperature with hemin (1∶32 protein:hemin molar ratio) and/or CaCl_2_ (1∶130 protein:Ca^2+^ molar ratio) before trypsin addition. Fragments were resolved by SDS-PAGE and visualized by Coomasie blue staining. Fragments were analyzed at the Virginia Tech Mass Spectrometry Incubator.

### Spectroscopic studies of TCTP-heme binding

Ten mM hemin (Frontier Scientific) stock solution was prepared in 50 mM Tris and 0.2 M KOH; and the pH adjusted to 7.8 using 1 M HCl. Hemin was added to glass cuvettes–one containing buffer (10 mM Tris, pH 8.0) and the other containing buffer and TCTP (5 µM) to a final volume of 500 µl. Visible scans were recorded between 300 and 700 nm on a Beckman DU-640 UV-VIS spectrophotometer (Beckman Coulter). Differences in heme absorption spectra were obtained by subtracting the buffer/hemin scan (blank) from each TCTP/hemin scan (sample). TCTP-heme binding was titrated with 0, 5, 10, 20, and 40 µM hemin and 5 µM TCTP.

For calcium titration, CaCl_2_ (Ca^2+^) was added to both a sample containing buffer and hemin (40 µM) and another sample containing buffer, TCTP (5 µM), and heme (40 µM). Differences in heme absorption spectra were obtained by subtracting the hemin/Ca^2+^ scan (blank) from the TCTP/hemin/Ca^2+^ scan. TCTP-heme binding was titrated with 0.04–1 mM of CaCl_2_.

### Cell transfection and pull-down assays

Chinese Hamster Ovary (CHO) cells were cultured in F-12K medium (Invitrogen) supplemented with 10% fetal bovine serum, penicillin (100 U/mL), streptomycin (100 µg/mL), gentamycin (50 µg/mL), and amphoterin B (250 ng/mL) and maintained at 37°C and 5% CO_2_. Cells were then transfected with 1 µg of pCS2+*myc*-TCTP using lipofectamine (Invitrogen) and cultured for an additional 12 h to allow expression of the TCTP protein. Cells were then cultured in serum-free medium containing 5 mM succinylacetone for 24 h prior to harvesting. Pellets were resuspended in lysis buffer (50 mM Tris-HCl, pH 7.5, 10 mM MgCl_2_, 200 mM NaCl, 1% NP-40, and 5% glycerol).

For pull-down assays, recombinant GST-TCTP bound beads were incubated with transfected CHO extracts in the presence and absence of hemin (at 1 µM, 100 µM, and 1 mM), and/or CaCl_2_ (2.5 or 25 mM) for 2 h at 4°C. Beads were washed with low and high stringency pull-down buffer A (10 mM sodium phosphate pH 7.5, 250 mM NaCl, 5 mM EDTA, and 0.1% Triton X-100) and B (same as A but with 1 M NaCl). Samples were analyzed by immunoblotting using α-myc and GST-specific antibodies (Santa Cruz).

## Results

In an attempt to consolidate some of the conflicting data surrounding the role of ligand binding in TCTP function, its influence in TCTP behavior, as well as the nature of their biochemical interaction in a comprehensive model, we evaluated the role of known interactors, such as heme and Ca^2+^, in modulating structural rearrangements associated with TCTP oligomerization and ligand binding.

### Ligand binding influences the oligomeric state of TCTP

Recombinant TCTP was expressed in *E. coli* as a GST N-terminus tag fusion protein, purified using an affinity column, and digested with thrombin. After chromatography, the untagged TCTP that was obtained was essentially pure as judged from the single band observed from Coomassie blue-stained polyacrylamide gels (data not shown). Protein preparations were subjected to mass spectrometry and N-terminus sequencing and shown to have an N-terminus in consonance with a proper signal peptidase cleavage.

**Figure 1:**
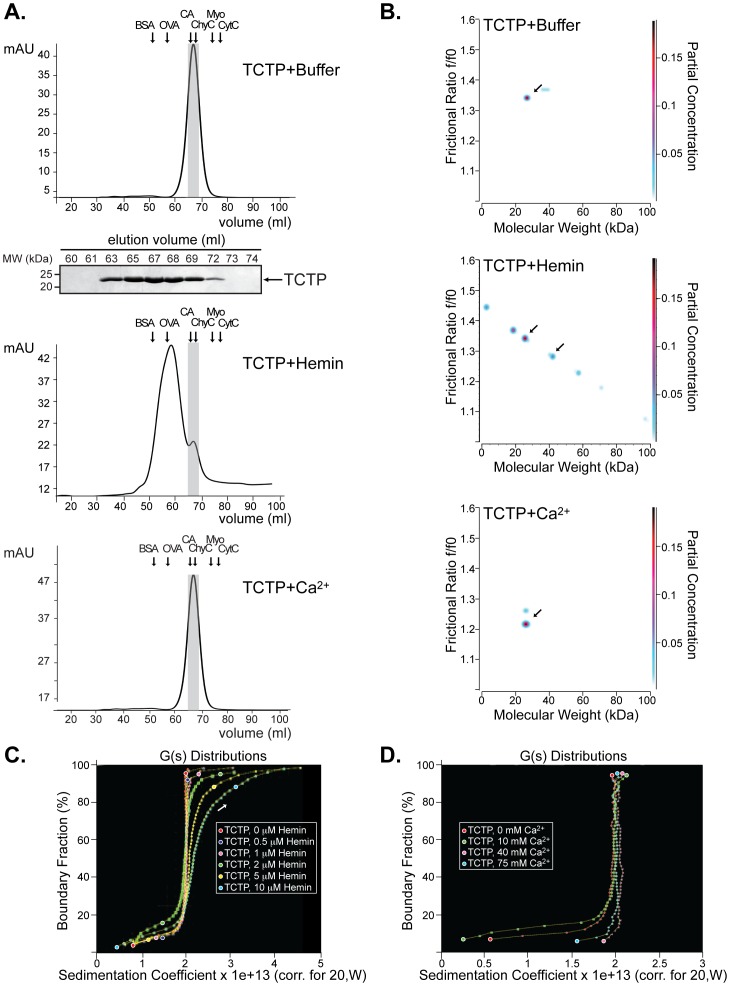
Ligand-binding influences TCTP oligomeric state. **A.** Elution profile of recombinant untagged-TCTP resolved by gel filtration using a 16/60 Superdex 75 column (60×1.6 cm) as described in “Materials and Methods” (*upper panel*). Aliquots of peak fractions were analyzed by SDS-PAGE and Coomassie blue staining (*inset panel*). In other experiments, untagged-TCTP was loaded onto a 16/60 Superdex 75 column pre-equilibrated with either 50 mM Tris-HCl, pH 7.8, 250 mM NaCl, and 1 mM hemin (*middle panel*) or 50 mM Ca^2+^ (*lower panel*). Peak fractions were analyzed as described above and shown in the *lower panel*. The calibration curve for the experiment was carried out using the following proteins as molecular mass markers: BSA: bovine serum albumin (67 kDa); OVA: ovalbumin (45 kDa); CA: carbonic anhydrase (29 kDa); Chy: chymotrypsinogen (25 kDa); Myo C: myoglobin (17 kDa); Cyt C: cytochrome C (12.5 kDa). Molecular mass markers (in kDa) are indicated on the left in each gel panel. **B–D.** Sedimentation velocity experiments of TCTP (0.3 µM) in the absence or presence of ligands (hemin and Ca^2+^). Genetic algorithm-Monte Carlo results (TCTP, *upper panel*; TCTP +10 µM Hemin, *middle panel*; TCTP +15 mM Ca^2+^, *bottom panel*), and integral van Holde-Weischet s-value distributions (+ hemin, *left panel*; + Ca^2+^, *right panel*) show increased oligomerization of TCTP in the presence of hemin but not Ca^2+^. In (**B**), the y-axis shows the frictional ratio (f/f_0_), which measures the globularity of the solute. An f/f_0_  = 1 indicates a spherical molecule. Partial concentration is color-coded and indicates optical density measured at 230 nm. The full sedimentation range is shown in (**C and D**).

Gel filtration chromatography of TCTP preparations eluted in a single and well-defined peak centered at an elution volume of 67.2 ml, which corresponds to an apparent mass of approximately 22 kDa, in close agreement with previous reports (Figure 1A, *upper panel*, [21]). Aliquots of peak fractions were analyzed by SDS-PAGE and a single band corresponding to the calculated MW of TCTP was detected (Figure 1A and *gel inset*; Figure S1A). To investigate the involvement of the heme prosthetic group in TCTP dimerization under native conditions, we analyzed the gel filtration profile of TCTP when loaded into a column pre-equilibrated with a buffer containing hemin (1 mM) and after being pre-incubated with the ligand for 15 min. As a result, the TCTP peak shifted with most of the protein eluting at 59.6 ml, a volume that corresponded to an estimated molecular mass of 45 kDa (Figure 1A, *middle panel*); thus, we conclude that TCTP is predominantly dimeric (named TCTP_d_ hereafter) under the conditions of this assay. Despite being symmetrical, the elution peak of TCTP in the presence of hemin was broad, which suggested a distribution of additional species in the solution [40]. The finding of a shoulder corresponding to the monomeric form of TCTP (named TCTP_m_ hereafter; Figure 1A, *middle panel*) was detectable under the conditions of the assay and was conspicuous in experiments where the concentrations of hemin were ≤700 µM (Figure S1B).

Calcium-binding activity constitutes a phylogenetically-conserved key attribute among TCTPs that is required to ensure appropriate intracellular Ca^2+^ levels and a constant extracellular Ca^2+^influx. As a result, we evaluated whether Ca^2+^levels influence TCTP oligomerization in a manner that closely resembles the effect of hemin in TCTP binding. To test this possibility, we chose to evaluate concentrations of Ca^2+^ from the nM-mM range that represent the broad levels of the ion found in the cytosol of eukaryotic cells (10–100 nM), lumen of endoplasmic reticulum (low 0.1–1 mM), and extracellular environment (>10 mM) [29], [41]. TCTP was resolved by gel filtration chromatography in the absence or presence of various Ca^2+^ concentrations (only the highest concentration is shown for simplicity, Figure 1A, *lower panel*); however, unlike hemin, concentrations of up to 50 mM of the ligand did not influence TCTP's oligomeric state and, thus, the protein remained as a monomer in solution.

The oligomeric structure of TCTP in solution and in the presence of its ligands was studied by analytical ultracentrifugation (AUC) using sedimentation velocity experiments as described [42]. The analysis of TCTP at 230 nm resulted in a very pure single species measurement with an apparent MW of 21–22 kDa (Figure 1B, *upper panel*). In addition, there was no concentration dependence, indicating no propensity to dimerize as a response to mass action within the range of concentrations tested (OD from 0.45 to 1.3, Figure S2A). Addition of hemin, in concentrations from 0.5 to 10 µM, caused oligomerization of TCTP that resulted, predominantly, in dimerization, but also in the formation of other minor oligomeric species as detected at 230 nm (Figure 1B, *upper vs. middle panels*). Conversely, calcium addition (up to 75 mM) did not cause any oligomeric shift in TCTP and the protein remained largely monomeric (Figure 1B, *lower panel*). Accordingly, velocity sedimentation data showed that the sedimentation coefficient of TCTP:hemin was indeed greater than that of TCTP alone or in the presence of Ca^2+^ (up to 75 mM; Figure 1D) for a range of hemin concentrations varying from 0.5 to 10 µM (Figure 1C), suggesting that TCTP:hemin can form a stable complex *in vitro*.

**Figure 2:**
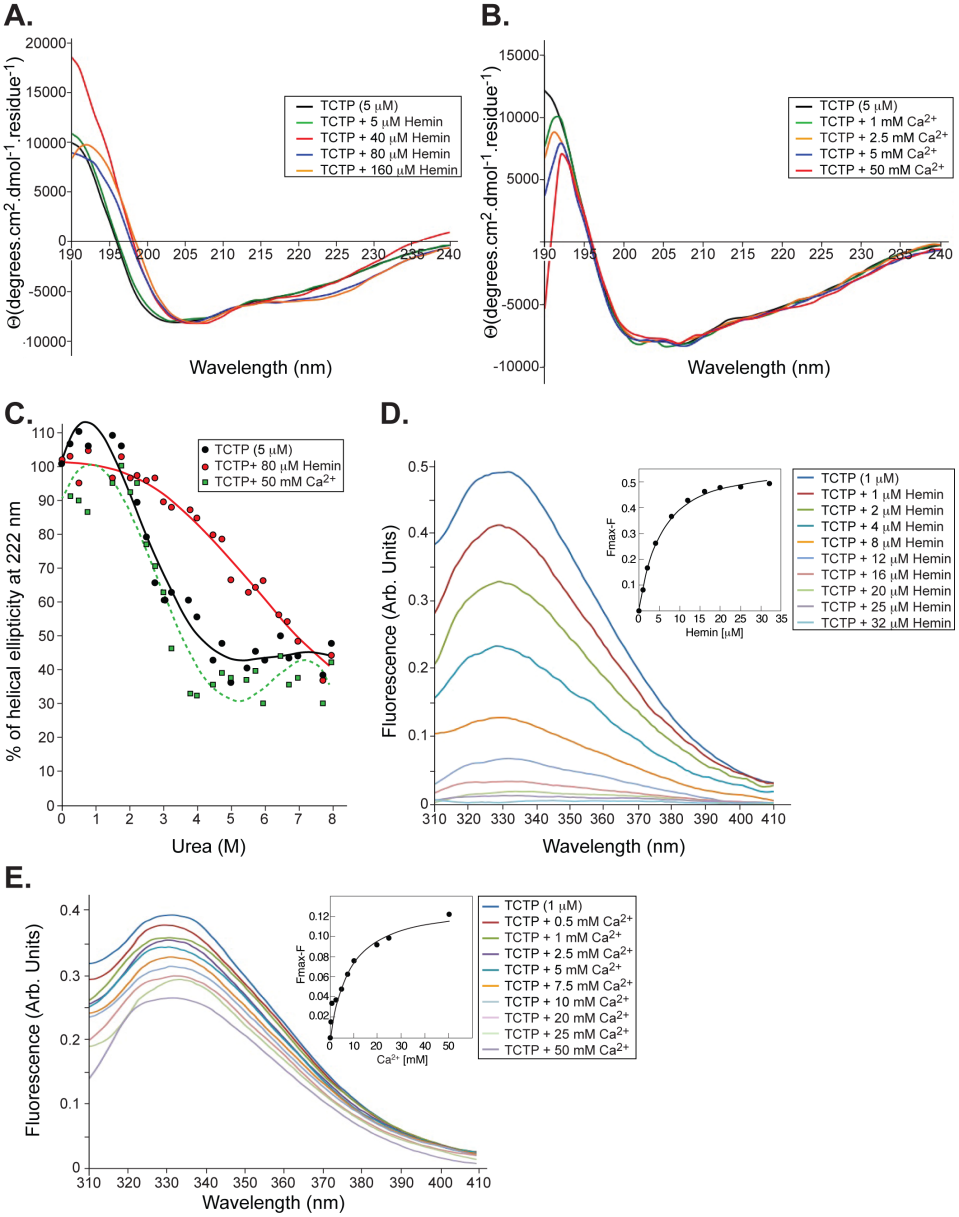
Binding of heme, but not Ca^2+^, influences TCTP conformation. **A.** Far-UV circular dichroism spectra of TCTP (5 µM, black line) in the presence of increasing concentrations of hemin (5 to 160 µM) at pH 6.8, 298°K. **B.** Circular dichroism spectra of TCTP (5 µM, black line) and in the presence of increasing concentrations of Ca^2+^ (1 to 50 µM) at pH 6.0, 298°K. **C.** Representation of the normalized fraction of folded molecules as a function of urea concentration. Urea-induced unfolding of TCTP alone (5 µM, •) and in the presence of either hemin (80 µM, red ○) or Ca^2+^ (50 mM, green □). **D.** Fluorescence spectra of TCTP (1 µM) in the presence of increasing concentrations of hemin (1 to 32 µM); pH 6.8, 298°K, λ_ex_ = 295 nm. Spectra are representative of three independent experiments. Inset: Plot of quenched fluorescence emission of TCTP (Fmax-F, where Fmax represents the maximum fluorescence level from hemin-free protein) *vs.* hemin concentration (from 1 to 32 µM). Plots were fitted using the nonlinear regression equation in Kaleidagraph. **E.** Fluorescence spectra of TCTP (1 µM) in the presence of increasing concentrations of Ca^2+^ (1 to 50 mM); pH 6.8, 298°K, λ_ex_ = 295 nm. Spectra are representative of three independent experiments. Inset: Plot of the reduction of the fluorescence emission of TCTP (Fmax-F, where Fmax represents the maximum fluorescence level from Ca^2+^-free protein) *vs.* Ca^2+^ concentration (from 0.5 to 50 mM). Plots were fitted using the nonlinear regression equation in Kaleidagraph.

Analyses of TCTP behavior in the presence of either hemin (40–640 µM) or Ca^2+^ (1–50 mM) under nondenaturing gel conditions resulted in oligomeric changes that were solely associated with hemin binding with no conspicuous effects as a result of Ca^2+^ addition (Figure 2B–C). Mass spectrometry sequencing of stained bands resulted in the identification of both N- and C-terminus peptides representing both the monomer and heme-containing dimer forms of TCTP (data not shown). Interestingly, TCTP_d_ was found to migrate faster than its monomeric form in native gels. Although unexpected, this is in agreement with previous observations in which, upon heme binding, some proteins exhibit an altered pI (∼0.4 units) followed by conformational changes (see next section) that influence their electrophoretic behavior [43], [44]. The existence of a dimeric form of TCTP in the presence of hemin was confirmed by BS3 crosslinking (Figure S2D). These additional results suggest that the buffering property of TCTP might be the result of a distinct structural reorganization upon ligand binding.

### Binding of hemin causes structural rearrangements in TCTP

We employed far-UV CD spectroscopy to analyze the effect of hemin on the secondary structure of TCTP. As expected, TCTP exhibited a CD spectrum with a minimum at ∼205 nm and a shoulder at 222 nm, characteristic of α/β proteins (Figure 2A). Changes in the CD spectrum were evident when hemin concentrations were over 40 µM (1∶4 protein:hemin ratio), evidenced by a shift of the spectrum minimum at 208 nm and a more negative minimum at 222 nm, indicating a gain of α-helical content. At higher hemin concentrations, the conformational state is stabilized and does not change further. On the other hand, addition of Ca^2+^ did not promote any changes in the CD spectrum of TCTP even at millimolar concentrations of the ligand (Figure 2B). Using the same experimental conditions, we monitored the mean residue ellipticity of TCTP at 222 nm as a function of urea concentration to follow the urea-mediated unfolding process (Figure 2C). The sigmoidal denaturation of the TCTP plot indicates that the unfolding process is cooperative, with an estimated [D]_50%_ of 3.9 M urea. TCTP pre-incubation with 80 µM hemin led to an enhancement in the stability of TCTP against urea-induced unfolding as indicated by the shifts of the unfolding curves to higher denaturant concentrations with an estimated [D]_50%_ of 6.8 M urea. In contrast, the presence of Ca^2+^ led to a less stable TCTP with a [D]_50%_ of 3.5 M urea.

### Binding affinities differ significantly among ligands

Next, we evaluated the ligand binding properties of TCTP to hemin and Ca^2+^ using intrinsic tryptophan fluorescence. In all cases, background spectra (blank buffer plus ligand; an example is shown in Figure S3A) were collected under similar experimental conditions and subtracted to obtain the final fluorescence spectra shown in Figure 2. Since TCTP lacks tryptophan residues, we introduced one by replacing the conserved Phe^129^ located in the loop between helices 4 and 5 with Trp. Despite this residue being close to the proposed noncanonical binding site for Ca^2+^ [27], there is no evident change on the overall secondary structure of TCTP Phe^129^Trp when compared with the wild-type protein (Figure S3B–C). The intrinsic fluorescence of this unique Trp is likely to be extremely sensitive to environmental perturbations around the amino acid residue and, thus, we would be able to monitor binding interactions as a measure of the protein fluorescence spectra upon addition of either Ca^2+^ or hemin to TCTP.

As can be seen in Figure 2D, addition of hemin from 1 to 32 µM led to quenching of Trp^129^ fluorescence in a concentration dependent manner with an estimated dissociation constant (*K*_D_) of 4.82±0.30 µM (χ^2^ 0.000651). The drop in tryptophan fluorescence can be explained by assuming conformational changes in TCTP as a result of ligand binding and quenching by other amino acid residues that were brought closer to Trp^129^ once the conformational change was triggered. These data suggest that hemin binds to a site close to that for Ca^2+^ with moderate affinity. As expected, addition of Ca^2+^ (0.5 to 50 mM) also triggered TCTP quenching spectra with a *K*_D_ of 8.02±1.16 mM (χ^2^ 0.000348) (Figure 2E). As Ca^2+^ was increased from 0 to 50 mM, fluorescence decreased gradually. At 10 mM, the intensity was reduced to about 25% of the initial value, and reached 65% reduction at higher concentrations. Neither hemin nor Ca^2+^ caused any obvious shift in the emission peak wavelength, thus, it is unlikely that increased hydrophobicity around Trp^129^ occurred.

### Spectroscopic analysis of heme-TCTP binding

**Figure 3:**
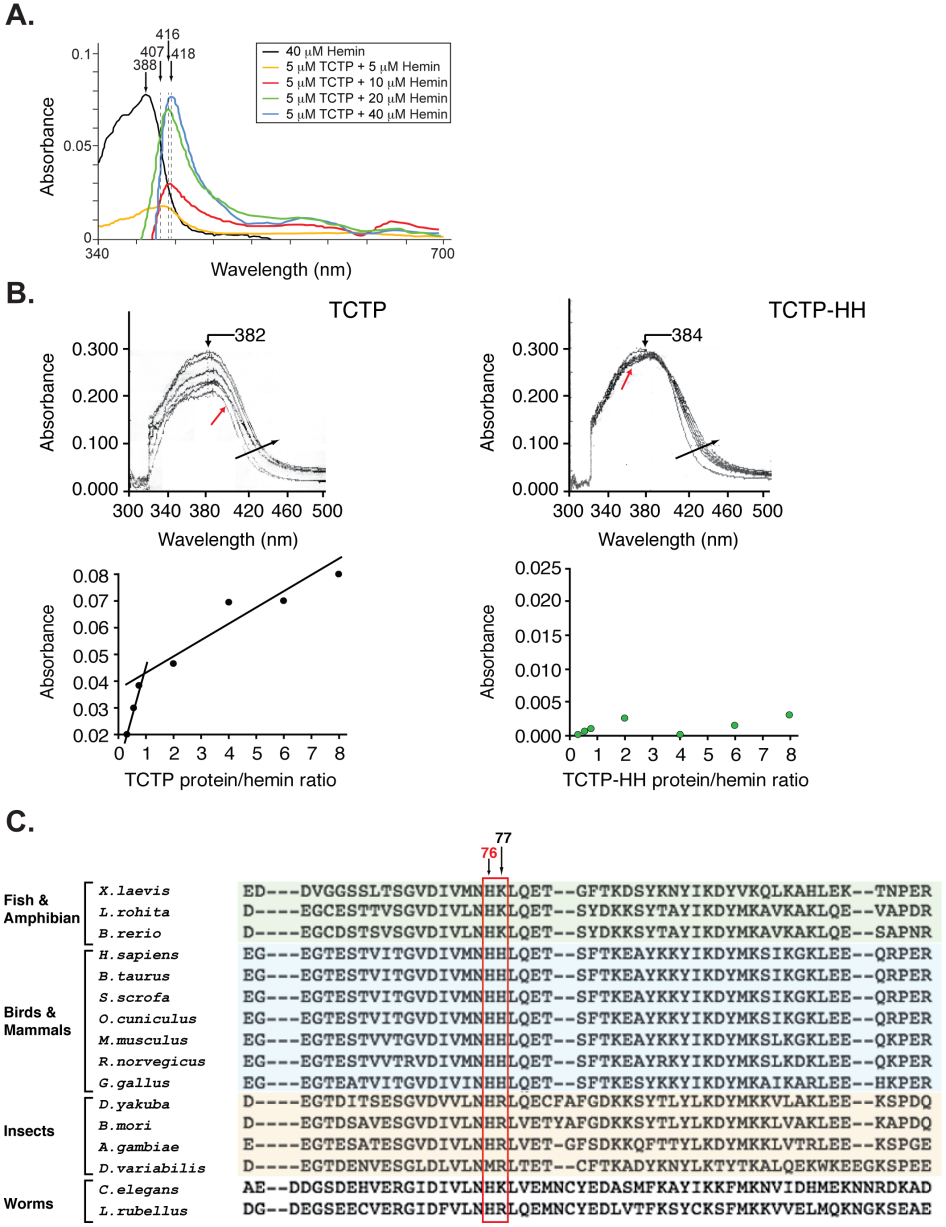
Heme binds TCTP in a conserved His residue. **A.** Absorption spectra of recombinant TCTP (5 µM) in the presence of increasing concentrations of hemin (5 to 40 µM). For each hemin concentration, the spectra for the latter were subtracted from the hemin alone (black line). **B.** Absorption spectra of hemin after adding increasing concentration of either TCTP (*top left panel*) or TCTP-HH (His^76^Ala and His^77^Ala, *top right panel*) protein up to 8 mol equivalent of the hemin amount (black arrow). Free hemin spectrum is indicated with a red arrow. *Bottom*: Titration curves of hemin are represented as absorbance at 382 nm (for TCTP) and 384 nm (for TCTP-HH) as a function of the molar ratios of the protein to hemin. **C.** Sequence alignment of the surrounding His 76 and 77 residues in human TCTP and its homologs in other species. Multiple alignments were performed with the program CLUSTALW and refined manually. The arrows on top indicate the position of His^76^ (red arrow) and His^77^ (black arrow). Sequences shaded in green, blue, and orange correspond to species with the fish and amphibian, birds and mammals, and insects, respectively. No shaded sequences are species from worms. Accession numbers for all species are listed in the “Materials and Methods” section.

To further examine the specificity of hemin interaction with human TCTP, we performed UV-visible absorption spectra experiments in which TCTP (5 µM) was titrated with increased concentrations of hemin (5 to 40 µM), which, if direct binding occurs, would result in a shift in the Soret peak to a different wavelength (Figure 3A). We chose a range of concentrations of hemin (<40 µM) for which there would be neither a contribution from oligomerization of TCTP nor a major structural change associated with a peak shift (Figures 1 and 2A). As shown, the TCTP-hemin complex exhibits a markedly different spectrum than hemin alone (388 nm) with an absorption maximum at 407 nm (for 5 µM TCTP) that grows as a result of increasing concentrations of hemin with a sharper Soret peak at 418 nm. The shift of the Soret peak to a longer wavelength excludes the possibility of direct bonding between the cysteine sulfur and the iron atom of heme [45], a prediction that was experimentally confirmed by absorption spectroscopy using two versions of TCTP in which each bear a mutation to Ala in either the Cys^28^ or Cys^172^ residues (data not shown). Moreover, the existence of a red-shifted Soret peak as a result of ligand addition is indicative of the involvement of either His/bis-His or His-Met as potential axial ligands for heme in TCTP. Remarkably, a His pair is present at positions 76 and 77 in human TCTP. The role of His^76^His^77^ in hemin-TCTP interaction was evident from titration experiments in which absorption spectra data was collected under conditions of increasing concentrations (1.25–40 µM) of either TCTP wild type or TCTP-HH (His^76^ and His^77^ were replaced by Ala) under a fixed concentration of hemin (5 µM). As seen in Figure 3B, addition of TCTP wild type (*left panel*), but not TCTP-HH (*right panel*), resulted in changes in the amplitude of the hemin peak that, when plotted as absorbance *vs.* protein/hemin ratio (*lower left and right panels*), defined the stoichiometry of TCTP-hemin interaction as equimolar. Altogether, these results demonstrate that hemin directly interacts with TCTP in, at least one distinct site (His^76^His^77^) but also that a larger exposed surface area in the protein more likely is involved in the recognition.

A close look at the multiple protein sequence alignment of TCTP in eukaryotes revels the existence of a conserved His-His motif across species that includes insects, worms, fish, amphibians, birds, and mammals (Figure 3C, [46]). Remarkably, His^76^ is conserved among all families with the exception of a single member, *D. variabilis*, in which His is replaced by Met, which is also a well-characterized axial ligand for heme. Unlike His^76^, position 77 seems to fluctuate among basic residues with His being absolutely conserved among birds and mammals but replaced by either Arg or Lys in other eukaryotes (Figure 3C). These observations prompt us to speculate that His^76^ might be the real axial ligand and that the surrounding conserved residues in positions 75, 77, and 78 (Asn^75^HisHisLeu^78^, with Asn and Leu strictly conserved in all eukaryotes) are likely to be required to further define the association.

In agreement with its putative role as an axial ligand, surface representation of human TCTP shows that both histidine residues have their side chains exposed to the solvent and, therefore, accessible for ligand binding (Figure S4, *panel i*). A molecular surface representation of the electrostatic potential surrounding the exposed histidine residues shows a predominantly negatively charged surface with a favorable electrostatic distribution for heme binding (Figure S4, *panel ii*). Lastly, we evaluated the relative importance of the different amino acid residues in the heme-binding interface by calculating the interface propensity for each residue type [47]. As shown in Figure S4 (yellow has higher propensity, *right panel*), residues described as favorable among heme-binding interfaces, including Cys, His, Met, Phe, Ile, Val, Trp, Tyr, and Arg [47], [48], in heme-containing proteins were highly represented in TCTP, further supporting the existence of a favorable interface for heme binding in the context of the His^76^ and His^77^ residues.

### Ligand-induced conformational changes in TCTP define structural regions needed for binding

**Figure 4:**
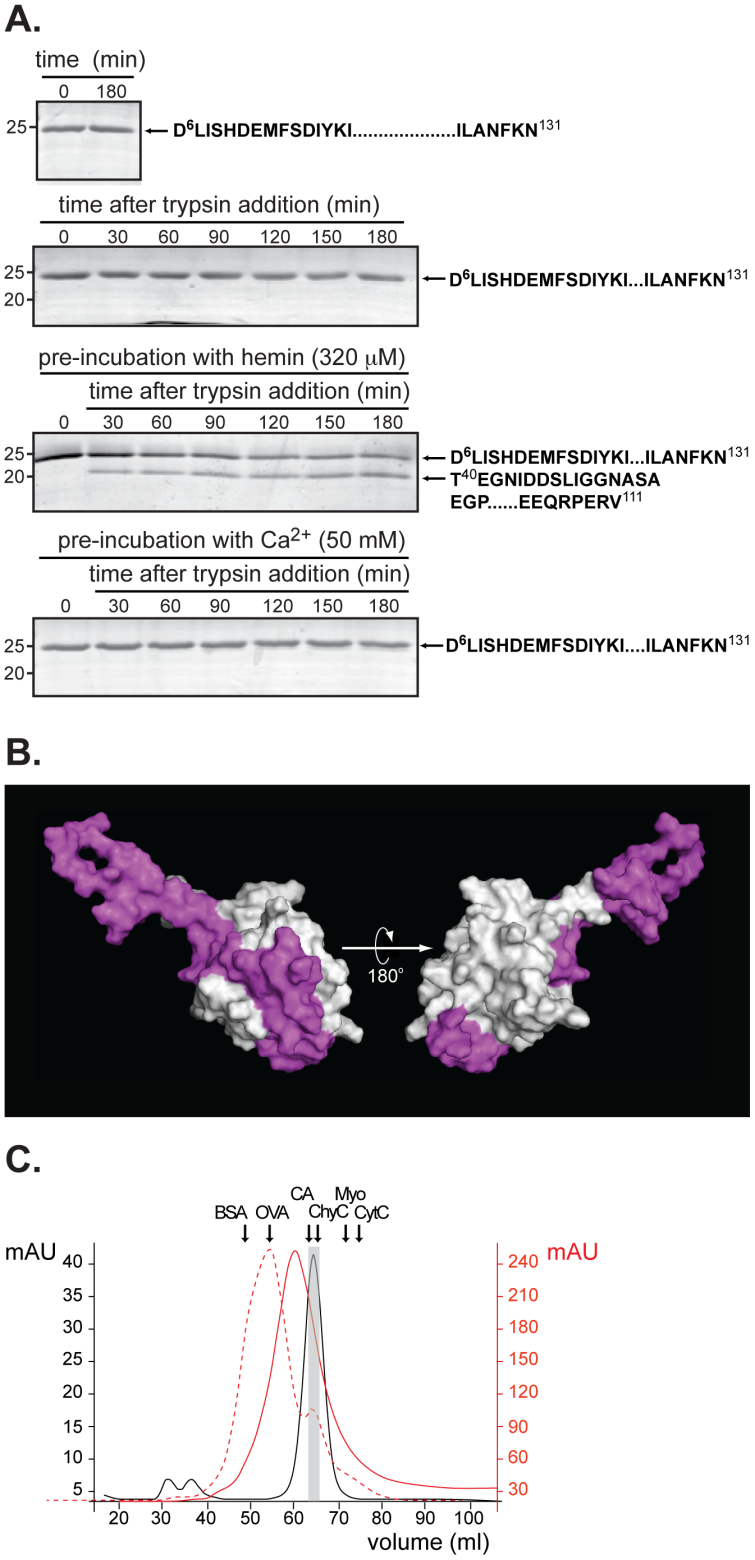
Limited proteolysis defines structural regions important for heme binding in TCTP. **A.** Recombinant untagged TCTP was pre-incubated, or not (*top panel*), with either hemin (320 µM, *middle panel*) or Ca^2+^ (50 mM, *bottom panel*) before the addition of trypsin as described in “Materials and Methods”. Stability of TCTP under digestion conditions was evaluated at room temperature throughout the time course analyzed (second *top down panel*). Samples were collected at indicated times and fragments resolved by SDS-PAGE and visualized by Coomassie blue staining. Molecular mass markers (in kDa) are indicated on the left. **B.** Two views of the surface representation of TCTP (PDB access code: 1YZ1) where the trypsin resistant fragment generated after hemin binding is displayed in magenta. **C.** Elution profile of recombinant untagged-TCTP-HH resolved by gel filtration using a 16/60 Superdex 75 column as described in “Materials and Methods” (solid black line). In other experiments, untagged-TCTP-HH was loaded onto a 16/60 Superdex 75 column pre-equilibrated with 50 mM Tris-HCl, pH 7.8, 250 mM NaCl, and 1 mM hemin (solid red line). Experiments were performed as in Figure 1 with untagged-TCTP in the absence or presence (dashed red line) of 1 mM hemin.

We further analyzed conformational differences in TCTP as a result of either hemin or Ca^2+^ binding using a limited-proteolysis approach. A time course analysis of trypsin-treated TCTP showed that this protein displayed remarkable stability retaining both N- and C-terminus epitopes as identified by mass spectrometry sequencing of stained bands (Figure 4A, *first and second panels from top*). Remarkably, pre-incubation of TCTP with hemin at a concentration known to induce TCTP dimerization resulted in increased susceptibility to trypsin cleavage (Figure 4A, *third panel from top*). Thus, the lower band reflected the cleavage of the N-terminus domain, as outlined by mass spectroscopy, within a region (Gly^40^-Gly^61^) defined as highly mobile and disordered in the TCTP structure [49] and that comprises the TCTP1 motif important for interactions [50]. When analyzed in the context of TCTP's structure, residues 40 to 111 are located within the same interface comprising the predicted heme-binding site suggesting that this interface might mediate dimerization (Figure 4B). Remarkably, pre-incubation of TCTP with Ca^2+^ (up to 50 mM) did not result in changes associated with ligand binding where putative trypsin sites might be exposed (Figure 4A, *bottom panel*). Identification of approximate sites of proteolysis was carried out using mass spectrometry (Table S1). TCTP sequencing resulted in the generation of peptides that covered 32% of its complete amino acid sequence when digested with trypsin and up to 48% when using four different proteases. The most N-terminus residue identified in trypsin-digested samples was Asp^6^, while Asn^131^ was identified as the last C-terminus residue in TCTP. A large part of the C-terminus domain of TCTP (residues 133 to 172) did not contain closely spaced trypsin cleavage sites and, therefore, likely generated peptide fragments (>3.5–4.0 kDa) too large for detection by mass spectrometry. In other cases, two putative trypsin sites within the C-terminus were too close (residues 168 and 171) and, thus, the peptide resulting from the digest might be too small (<800 Da) for detection. Of note, we identified two peptides (^63^E.STVITGVDIVMNHHLQE.T^81^ and ^102^K.LEEQRPER.V^111^) by mass spectroscopy that had a high affinity for iron with one of them containing the putative bis-His axial ligand for heme binding, which was described as a potential binding site in the previous section (residues His^76^ and His^77^ underlined in the sequence). Overall, these results suggest that the increased proteolytic susceptibility is accompanied by TCTP reorganization upon hemin binding.

Next, we evaluated whether mutation in residues His^76^ and His^77^ to Ala influence TCTP dimerization (Figure 4C). Gel filtration chromatography studies were carried out in the absence (solid black line) or presence of hemin (1 mM, solid red line) for TCTP-HH and compared to the protein profile obtained for the wild type protein in the presence of the ligand (1 mM, dashed red line). Like TCTP, TCTP-HH behaves as a single monomer in the absence of ligand as determined by comparing the peak volume at which both proteins elute. TCTP and TCTP-HH were then pre-incubated with hemin and resolved by chromatography using a column pre-equilibrated with the ligand. Unlike TCTP, TCTP-HH was unable to form dimers with hemin (red solid *vs*. dashed red lines); however, a minor shift in the elution peak was observed (black *vs.* red solid lines). We speculate that this might reflect non-specific binding of the ligand to the interface of the protein that comprises residues exhibiting high propensity values (Figure S4, panel *iii*). This might, in turn, result in the accumulation of multiple forms of the protein-ligand complex for which a width peak is observed.

### Calcium modulates heme-mediated dimerization of TCTP

TCTP was identified as a Ca^2+^ binding protein using deletion constructs, binding overlay assays, and NMR studies [26], [27]. As a result, a low-affinity Ca^2+^ site was predicted to be located within residues 81–112 of TCTP [26], although chemical shift perturbations were observed in residues His^77^, Tyr^151^, Tyr^132^, and Gln^133^ by NMR [27]. Because the Ca^2+^ binding site seems to be proximal to that of the heme in TCTP, we evaluated the possibility of being able to displace heme from binding to TCTP and influencing its oligomerization state.

**Figure 5:**
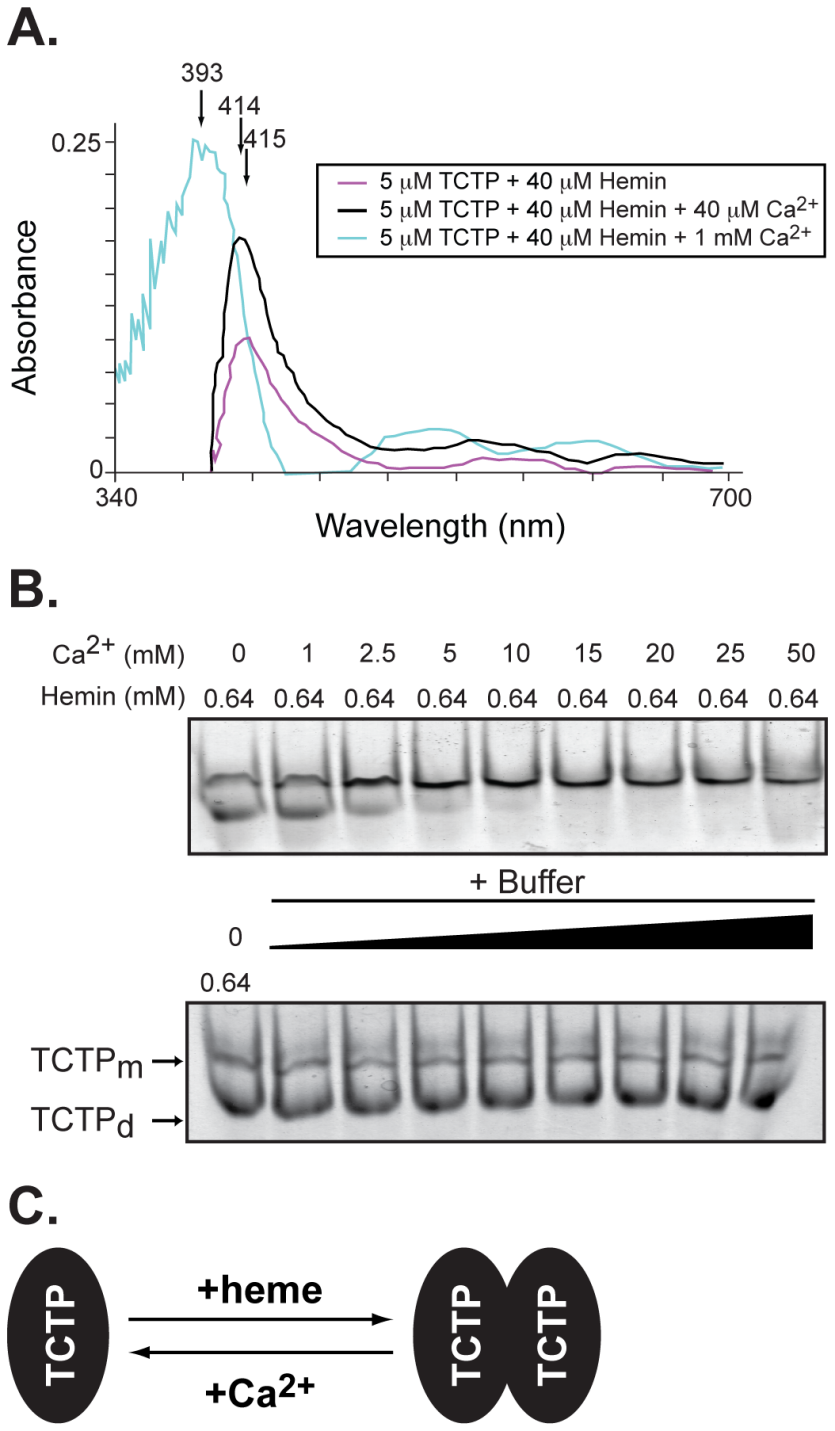
Calcium binding displaces hemin and influences TCTP oligomerization. **A.** Absorption spectra of recombinant TCTP (5 µM) in the presence of hemin (40 µM) and as a result of the addition of increasing concentrations of Ca^2+^ (40 µM and 1 mM). For TCTP-hemin, the spectra were subtracted from the hemin alone. In addition, all TCTP/hemin/Ca^2+^ samples were subtracted from the hemin/Ca^2+^ alone. **B.** Electrophoretic profile of TCTP separated by native gel electrophoresis and visualized by Coomassie blue staining. In all cases, TCTP (8 µg) was pre-incubated with hemin (640 µM, left lane in each panel) before the addition of increasing concentrations of Ca^2+^ (1 to 50 mM, *left panel*). Buffers were used as controls (*right panel*). **C.** Cartoon representation of ligand interplay in TCTP oligomerization.

Absorption spectra studies were carried out in the presence of pre-bound TCTP/Hemin (1∶8 ratio, 5 µM of protein) and increasing concentrations of Ca^2+^ (Figure 5A). As shown in Figure 5A, equimolar concentrations of hemin and Ca^2+^ did not cause a major shift in the Soret peak that corresponds to the TCTP/Hemin bound complex. This is most likely due to the low affinity of the Ca^2+^ binding site as seen by a shift to the blue of the Soret peak when Ca^2+^ concentration was increased to 1 mM (with hemin:Ca^2+^ ratio going from 1∶1 to 1∶25). As a result, it seems plausible that high concentrations of Ca^2+^ would disrupt the heme/TCTP interaction and therefore affect the stability of the TCTP_d_ complex.

We then asked whether competition of hemin binding to TCTP by Ca^2+^ causes changes in the oligomeric state of the protein. To test this possibility, we evaluated the mobility shift of TCTP in native gels when bound to hemin and compared it to that obtained in the presence of increasing concentrations of Ca^2+^ (Figure 5B). Results show that Ca^2+^ addition directly impacts dimer formation, an effect that becomes evident when Ca^2+^ concentration increases at least 4-fold above hemin levels. In support of these findings, we found that when pooled fractions corresponding to the hemin-induced dimeric form of TCTP were incubated in the presence of Ca^2+^ (50 mM) and re-run by gel filtration, the peak shifted back to mainly a monomeric state indicating that, within this range of concentrations, TCTP predominantly exists as a mixture of monomers and dimers in a rapid, dynamic equilibrium (Figure S5). Overall, our data show a direct regulation of TCTP oligomerization by ligand binding with hemin promoting a dimerization state, whereas Ca^2+^ stabilizes the TCTP monomer state (Figure 5C).

### NMR Studies

**Figure 6:**
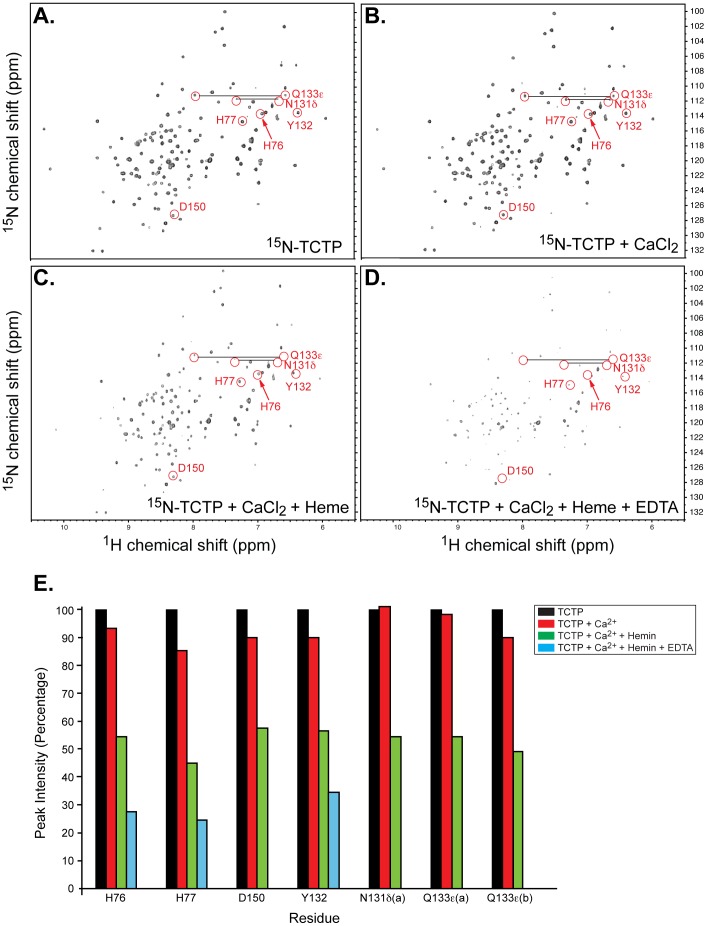
NMR spectroscopy supports ligand binding interplay. Two-dimensional ^1^H, ^15^N HSQC NMR spectra of ^15^N-labeled TCTP (200 µM) in the absence (**A**) and presence of CaCl_2_ (5 mM) (**B**), CaCl_2_ (5 mM) and hemin (1.6 mM) (**C**), and CaCl_2_, hemin, and EDTA (10 mM) (**D**). Resonances that line broadened are circled and labeled with the corresponding TCTP residue. **E.** Quantification of chemical shifts' intensities of indicated TCTP residues from the HSQC spectra shown in panels **A–D**.

To confirm and further characterize heme interaction at atomic resolution, we collected two-dimensional HSQC spectra of ^15^N-labeled TCTP. The ^1^H-^15^N HSQC spectrum of TCTP displayed good chemical shift dispersion of its ^1^H-^15^N resonances, indicative of a folded structure (Figure 6A). The narrow line widths of the resonances suggested that TCTP was in a monomeric state.

Next, hemin was titrated into the ^15^N-labeled TCTP, and hemin-associated chemical shift perturbations were monitored in HSQC spectra (Figure 6B–C). Although minor, hemin-induced chemical shift perturbations were reproducible and occurred in a concentration-dependent manner (data not shown). Addition of 8-fold excess of hemin led to both line broadening and perturbations of the TCTP NMR resonances (Figure 6C). Interestingly, these resonances were also perturbed by Ca^2+^ [27], suggesting that hemin and Ca^2+^ share common TCTP binding residues. The loss of resonance intensity is likely due to hemin-induced TCTP dimerization. Indeed, addition of EDTA to a hemin-enriched TCTP sample led to a significant decrease in resonance intensity possibly due to chelation of the remaining Ca^2+^ bound to the protein (Figure 6D), making more TCTP available for hemin binding. Major perturbations were in the His^76^, His^77^, Tyr^132^, and Asp^150^ backbone resonances as well as in the Asn^131^δ and Gln^133^ε side chain resonances (Figure 6E).

### TCTP oligomerization is competed by ligand binding in cells

**Figure 7:**
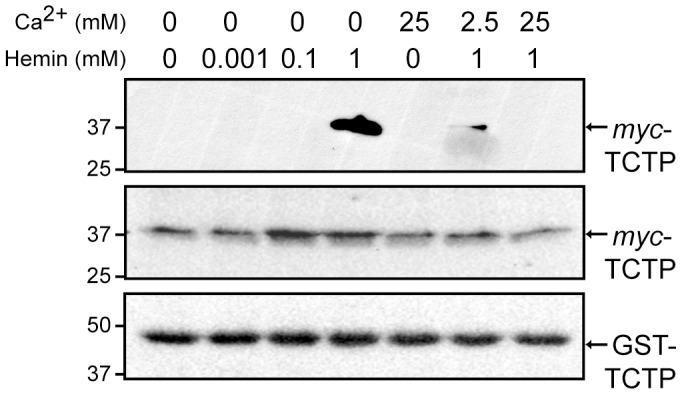
Cellular TCTP oligomerization is ligand-dependent. CHO cells were transfected with pCS2+*myc*-TCTP in serum-free medium containing 5 mM succinylacetone for 24 h prior to harvesting to prevent *de novo* synthesis of heme. Extracts were incubated with recombinant GST-TCTP bound beads in the absence or presence of hemin (1 µM, 100 µM, 1 mM), and/or CaCl_2_ (2.5 or 25 mM) and described in “Materials and Methods”. Bound complexes were resolved by SDS-PAGE and bound proteins detected by immunoblotting (*upper panel*). The expression of recombinant *myc*-TCTP in cells and GST-TCTP in the assay are shown in the *middle and lower panels* for each treatment, respectively. Molecular mass markers (in kDa) are indicated on the left.

We then asked whether endogenous TCTP would be able to form oligomers as predicted by our *in vitro* studies. As a result, we expressed a *myc*-tagged form of TCTP in CHO cells maintained in serum-free medium containing succinylacetone (SA), an inhibitor of the second enzyme (δ-aminolevulinic acid dehydratase) of the heme biosynthetic pathway. Treatment of CHO cells with SA led to a progressive decline in the endogenous heme concentration and, based on our model, should result in accumulation of monomeric TCTP. Extracts from SA-treated cells were then incubated with GST-bound TCTP to detect *myc*-TCTP binding in the presence of various ligand concentrations. Accordingly, GST-TCTP was only able to dimerize with endogenous *myc*-TCTP when the concentration of hemin added to the reaction surpassed the K_D_ value by several fold (Figure 7). In agreement with our *in vitro* findings, Ca^2+^ alone did not promote TCTP dimerization and increasing concentration of this ligand competed off *myc*-TCTP bound to GST-TCTP in the presence of hemin. Overall, our results support a model where TCTP helps maintain cellular homeostasis by acting as a buffer molecule that sequesters the unwanted excess of ligand in a soluble oligomeric form.

## Discussion

The functional importance of TCTP arises from the plethora of cellular processes in which it is involved and which span from its regulation of cell cycle and death processes at the intracellular level to its role in response to allergic inflammation when acting extracellularly (for review see [1]). As a result, the general concept is that TCTP exerts a cytoprotective function in the cell and a cytokine-like activity in the immune response. To add to TCTP's complex regulation, numerous stimuli and conditions control its level and influence on localization transitions, making this protein an attractive therapeutic target.

Our studies focus on the role of TCTP as a ligand binding protein and, thus, we propose a “*buffer-like*” function for TCTP that helps cells balance intra- and extracellular levels of specific ligands under physiological or pathological conditions. Two of the ligands we explored in our work were Ca^2+^ and heme. We chose these ligands because *i*) binding of both of them to TCTP was confirmed and, to some extent, studied [21], [26], [51], *ii*) altered cellular concentration of Ca^2+^ (mM range) correlates with changes in TCTP accumulation [4], *iii*) inhibition of heme binding to TCTP promises alternative routes for disease treatment [21], [52], and *iv*) both ligand levels play a role under physiological conditions and in disease development and progression [41], [53]–[55]. Thus, we hypothesized that TCTP displays several structural strategies to sequester excess ligand and help buffer conditions that otherwise would be detrimental to the cell.

Self-interaction among TCTP homologues was initially uncovered using a yeast two-hybrid system [56] and is now speculated to be an essential property for TCTP cytokine-like activity and in allergic inflammation [20], [24]. Attempts to define the binding region through which TCTP dimerization occurs has resulted in an accumulation of inconsistent data, none of which explains the need for dimerization when present in serum. For example, a construct of TCTP truncated on its N-terminal 35 residues dimerizes *in vitro*, increases the secretion of IL-8 and GM-CSF from BEAS-2B cells, and enhances TCTP allergic response as measured by inhibition of IL-2 and release of IL-4 from CD4^+^ T_H_ cells [20]. In this case, it is proposed that dimerization of truncated TCTP is mediated by an intermolecular disulfide bridge provided by the C-terminal Cys^172^ [20]. However, whereas structural studies might provide indirect support for this model, the biochemical data seem conflicting. Accordingly, the solution structure of *S. pombe* TCTP, which on the basis of sequence homology defines the fold of the entire family, closely resembles features of the Mss4/Dss4 family of guanine nucleotide exchange factors [50]. In this structure, both the N- and C-termini of TCTP are packed together as antiparallel β-sheets and, thus, it has been proposed that the N-terminus might interfere in the formation of disulfide bonds making its cleavage a requirement for dimerization to occur [50]. However, the same group later found that dimerization of full-length TCTP, rather than the truncated form, is essential for the activation of TCTP-mediated allergic response [20]. Because secretion of IL-8 was measured *in vitro*, the authors needed to artificially generate a dimeric form of TCTP for the study. This is an important detail when analyzed in the context of our studies since, we propose that dimerization of TCTP only occurs when high µM concentrations of heme are present, as is the case in serum but not in *in vitro* experiments unless specifically added (Figure 1). Consistent with our results is the finding that dimeric full-length TCTP can be readily detectable in sera from atopic or atopic/asthmatic patients [20].

Further support for a model of noncovalent dimerization of TCTP came from biochemical studies of secreted TCTP obtained from various extracellular environments. First, purified TCTP from bronchoalveolar lavage fluids was shown to be stable as a dimer when maintained in a reducing buffer. Second, dimers of TCTP are detected even when Cys^172^ of TCTP is replaced by Ser, and lastly, the activity of a dimeric form of TCTP generated by N-terminus cleavage is only partially abrogated under reducing conditions [20]. One more piece of evidence relates to findings that both *B. malayi* and *W. bancrofti* TCTPs form dimers under non-reducing conditions through a coiled coil structure in their sequence that comprises residues 92–124 [57].

Although the information summarized above seems controversial at first, it can be consolidated into one model based on our observations. We propose that heme binding to TCTP causes a conformational change that exposes the N-terminus of the protein and allows for two molecules of TCTP to interact by non-covalent bonding. This model largely relies on our findings that *i*) hemin promotes TCTP dimerization (Figure 1), *ii*) binding of hemin induces a conformational change in TCTP (Figure 2A) that results in a more stable complex as shown by urea denaturation assays (Figure 2C), *iii*) structural rearrangements allow for modification in the N-terminus that is now exposed and susceptible to trypsin cleavage (Figure 4B), *iv*) leaving a stable fragment comprising residues 40 to 111 that localize within the same interphase (Figure 4B). Moreover, our biochemical studies indicate a relatively low K_D_ for heme binding (∼5 µM) in TCTP (Figure 2D). Although binding occurs at low µM concentrations (Figures 2D and 3A), dimerization seems to occur at concentrations of heme roughly ten times higher (Figure 1). We propose that this serves a buffer purpose in different scenarios. For example, an excess of intracellular free heme promotes the formation of reactive oxygen species (ROS) that result in augmented oxidative stress and cell death [58]. Thus, we speculate that TCTP might act by complexing intracellular free heme and keeping it in a soluble, non-toxic, condition allowing the cell to effectively control the production of unwanted ROS. In this scenario, dimerization is unlikely to happen, and is not even needed, since endogenous concentration of free heme does not surpass the low micromolar range [59].

On the other hand, dimerization at high heme concentrations might be a physiological advantage under certain conditions in which sequestering heme and triggering an inflammatory response are both needed to help the body deal with a pathological condition. Examples of this scenario are in pathologies where there is a deficiency in expression or activity of hemo-oxygenase 1, the enzyme responsible of heme catabolism. As a result of this defect, cell damage leads to consistently high concentrations of heme in serum (∼0.5 mM, [60], [61]), a phenotype that is usually accompanied by various oxidative and inflammatory complications [60], [61]. Correlation between profound inflammatory responses and unscheduled accumulation of free heme in serum are known to accompany other pathological conditions, such as hemorrhage and hemoglobinopathies [62].

Next, we attempted to identify the putative binding site for heme in TCTP to find, unlike what was previously reported [20], that binding is mediated by a His coordination (Figure 3A–B). We largely based our conclusions on the fact that *i*) absorption spectra data show a Soret band shift towards the red as a result of hemin addition to TCTP, supporting His as an axial ligand (Figure 3A), *ii*) specificity of binding was shown by absorption spectra and titration experiments, in which addition of increasing amounts of protein results in increasing amplitude of the peak, an effect that is abrogated when His^76^ and His^77^ are mutated to alanine (Figure 3B, upper panels), *iii*) a two-dimensional representation of the titration data exposes a well-defined inflection point corresponding to a molar stoichiometry of hemin:TCTP of 1∶1 (Figure 3B, lower panels); accordingly, specific binding of hemin to TCTP His^76^Ala and His^77^Ala was not detected, *iv*) either His residue may act as the proximal histidine as mutations in both His^76^ and His^77^ residues are needed to completely abrogate hemin binding (data not shown), and *v*) since TCTP does not exhibit any appreciable absorption spectra between 300 and 700 nm, changes in the absorption spectra as a result of hemin addition are due to alterations in the electronic structure and coordination state of the heme iron caused by its interaction with TCTP.

The solution structure of human TCTP was determined by NMR spectroscopy and closely resembles that of *S. pombe* p23^fyp^ with a rigid well-folded core and a flexible long loop including the TCTP2 and TCTP1 regions, respectively, connected by two short β-sheets [27], [50]. Histidine residues 76 and 77 are exposed to the solvent and localize in a well-conserved, small helical feature (α1) found among TCTPs and between TCTPs and Mss4 (Figure 4A and [50]). In agreement with their binding role, both His residues are embedded in a heme-binding interface as defined by the ratio between the amino acid frequency in the heme binding interface and that in the rest of the protein that favors ligand interaction (Figure 4B, right panel and [47]), further supporting our findings.

Calcium plays a relevant role in TCTP biology by regulating its expression at the transcriptional and post-transcriptional levels in response to changes in Ca^2+^ concentration in different cellular compartments [4]. More recently, binding of Ca^2+^ to TCTP has been predicted to be of very low affinity based on solution structure studies [27]. Our data defines this ligand-protein interaction further and establishes a range of association in the lower millimolar value (Figure 2E). Furthermore, our findings prove that, unlike heme association, binding of Ca^2+^ to TCTP neither promotes TCTP's oligomerization (Figure 1A–B, and D) nor changes the protein's overall structure (Figures 2B and 4A). In agreement with a predicted “*buffer-like*” function for TCTP and its characterization as a non-traditional calcium-binding protein [27], TCTP localizes in both the cytosol and lumen of the endoplasmic reticulum in several normal cells and in tissues where it can serve to maintain the homeostatic balance of nM to mM Ca^2+^ levels present in these compartments [9], [29]. An example of the critical role that TCTP plays in buffering high concentrations of intracellular Ca^2+^ arises in its role in syncytiotrophoblasts, a group of cells responsible for transplacental transport of nutrients between mother and fetus. In these cells, low-affinity Ca^2+^ binding proteins, including TCTP, regulate the concentration of intracellular free Ca^2+^ available for active transport to fetal blood, which is the source of almost 80% of the total Ca^2+^ present in fetal circulation within the first trimester [63]. For this to be accomplished, syncytiotrophoblasts need to maintain a level of intracellular Ca^2+^ that is ∼1000-fold higher than other trophoblast cells, a level that is accomplished by sequestering cytosolic Ca^2+^ in low-affinity calcium binding proteins [28], [64].

Identification of any recognizable Ca^2+^ binding motif within TCTP is not conspicuous from a sequence analysis. The Ca^2+^ binding domain in *R. norvegicus* TCTP was first confined within residues 81–112 using ^45^Ca^2+^-overlay assays, a region that is mainly constituted of random coil [26]. However, structural studies suggest that the Ca^2+^ binding region should include a larger portion of the C-terminus end of TCTP as β-sheets A and B and that the α2–α3 helix-hairpin is absolutely required to maintain the appropriate folding and binding capacity of the 81–112 fragment [27]. Other specific residues found to be important for Ca^2+^ binding include Asn^131^, Tyr^132^, Gln^133^, Asp^150^ as shown in Figure 6 and [27].

Because heme and Ca^2+^ binding occur within the same interface in TCTP, we explored the effect of Ca^2+^ on the heme environment by absorption spectra in TCTP dimerization under native conditions (Figures 5 and 6). Crosstalk between Ca^2+^ and heme binding has been observed under various scenarios in the cell. For example, Ca^2+^ influences the orientation of residues within the heme-binding pocket in horseradish peroxidase C [65], [66]. It is also involved in structural changes within the heme macrocycle and its substituents in cytochrome *c* peroxidase [67] and is associated with the maintenance of the three dimensional structure of heme-containing enzymes [68], [69]. Our data show that Ca^2+^ addition displaces heme from TCTP and promotes dimer to monomer transition (Figure 5). The relevance of Ca^2+^-mediated oligomerization in heme-containing proteins is evident among cytochrome *c* peroxidases. Unlike human TCTP, Ca^2+^ influences *P. aeruginosa* cytochrome *c* peroxidase oligomerization by binding its interface and promoting dimerization, an essential step for activation [67]. In this case, Ca^2+^ occupancy is proposed to be pH-dependent although the mechanism of activation still remains elusive. Calcium binding in cytochrome *c* peroxidase also occurs in a second site located between the two heme ligands in the monomer; however, unlike the first site, the latter is always occupied and, therefore, pH-independent [70]. Lastly, the interplay among ligands and their role in TCTP dimerization in cells became evident when cells were treated with either ligand or a combination of them and TCTP's capacity to form oligomers was evaluated (Figure 7).

In conclusion, our findings establish the need for a conformational change associated with heme binding and TCTP oligomerization as a strategy to sequester potential deleterious excess of free heme in the cell and its environment when secreted. Although any definitive function for the TCTP-heme is a matter of speculation, there are additional possibilities. For example, TCTP might serve as either storage reservoir of heme or cellular sensor of heme availability for purposes of cellular regulation rather than heme scavenger. Moreover, TCTP's ability to respond to elevated levels of Ca^2+^ makes this unconventional Ca^2+^-binding protein a versatile biological switch capable of intervening in various signals. As a result, our model sheds light on the elusive behavior of TCTP in both intra- and extracellular compartments.

## Supporting Information

Figure S1 **A.** Full image of the gel corresponding to the TCTP purification shown in Figure 1A. Elution fractions are indicated on top. **B.** Elution profile of recombinant untagged-TCTP resolved by gel filtration using a 16/60 Superdex 75 column equilibrated with either 350 µM or 700 µM hemin (*upper and lower panels*, respectively) as described in “Materials and Methods”. Shaded box indicates the position of the monomeric form of TCTP. Red arrow indicates additional oligomeric forms present in the sample.
(TIF)

Figure S2 **A.** van Holde – Weischet G(s) distribution of TCTP species at two different concentrations (ODs 045 and 1.3). **B–C.** Electrophoretic profile of TCTP separated by native gel electrophoresis and visualized by Coomassie blue staining. In all cases, TCTP (8 µg) was pre-incubated with various concentrations of either hemin (**B**; 40 to 640 µM) or Ca^2+^ (**C**; 1 to 50 mM) before being loaded onto the gel. In each scenario, buffers were used as controls. **D.** Chemical crosslinking of GST (positive control), TCTP (*left panel*), or TCTP-HH (*right panel*; 5 µg each) and either hemin (H; 32 µM) or CaCl_2_ (Ca^2+^; 50 mM) in the presence (+) or absence (-) of BS3 as described in “Materials and Methods”. Right panel: GST and TCTP-HH were tested with two different concentrations of BS3 (1 and 3 mM) and samples were resolved by SDS-PAGE. Arrows indicate dimeric complexes.
(TIF)

Figure S3 **A.** Background spectra for the various concentrations of hemin (1–32 µM) tested in Figure 2D. **B.** Far-UV circular dichroism spectra of TCTP (5 µM, black line) and TCTP Phe^129^Trp mutant (5 µM, red line) at pH 6.8, 298°K. **C.** Predicted secondary structure content of TCTP and TCTP Phe^129^Trp mutant using the CDSSTR algorithm. R and D represent regular and distorted secondary structure elements, respectively. NRMSD: normalized root mean square.
(TIF)

Figure S4 **Surface representation of TCTP (PDB access code: 1YZ1) depicting the position of residues His^76^ and His^77^ (in cyan, panel** ***i*** **) and colored according to electrostatic charges (negative and positive potentials in red and blue, respectively; panel** ***ii*** **) and conservation scores based on interface propensity to bind heme shown in panel** ***iii*** **** [47] **.**
(TIF)

Figure S5 **Elution profile of recombinant untagged-TCTP pooled from fractions 65–68 from** **Figure 1A** **and resolved by gel filtration using a 16/60 Superdex 75 column pre-equilibrated with 50 mM CaCl_2_.** Red arrow indicates additional oligomeric forms present in the sample.
(TIF)

Table S1 **Summary of heme-protected TCTP fragments detected by MS/MS.** Results were obtained by digestion with individual proteases followed by MS/MS analysis. Protease are indicated with a one letter code and are: G: endoproteinase GluC; L: endoproteinase LysC; C: Chymotrypsin; T: Trypsin.
(DOCX)
