# Supplementary figures and images for: Ligand Binding Reveals a Role for Heme in Translationally-Controlled Tumor Protein Dimerization
Source: PLoS One. 2014 Nov 14;9(11):e112823. doi: 10.1371/journal.pone.0112823 (PMC4232476; doi:10.1371/journal.pone.0112823)

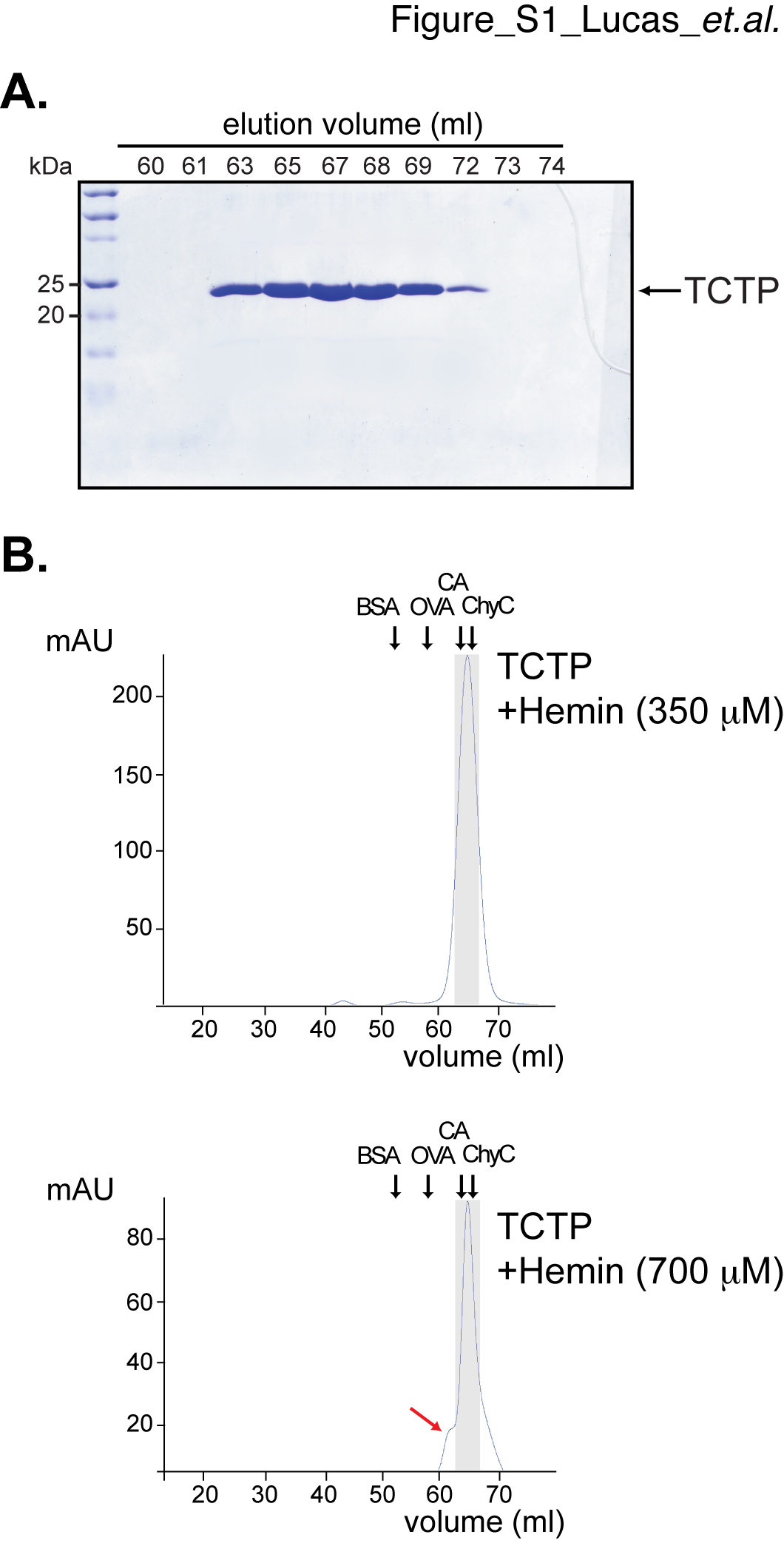

Supplement: Figure S1 — A. Full image of the gel corresponding to the TCTP purification shown in Figure 1A. Elution fractions are indicated on top. B. Elution profile of recombinant untagged-TCTP resolved by gel filtration using a 16/60 Superdex 75 column equilibrated with either 350 µM or 700 µM hemin (upper and lower panels, respectively) as described in “Materials and Methods”. Shaded box indicates the position of the monomeric form of TCTP. Red arrow indicates additional oligomeric forms present in the sample. (TIF) [file pone.0112823.s001.tif]

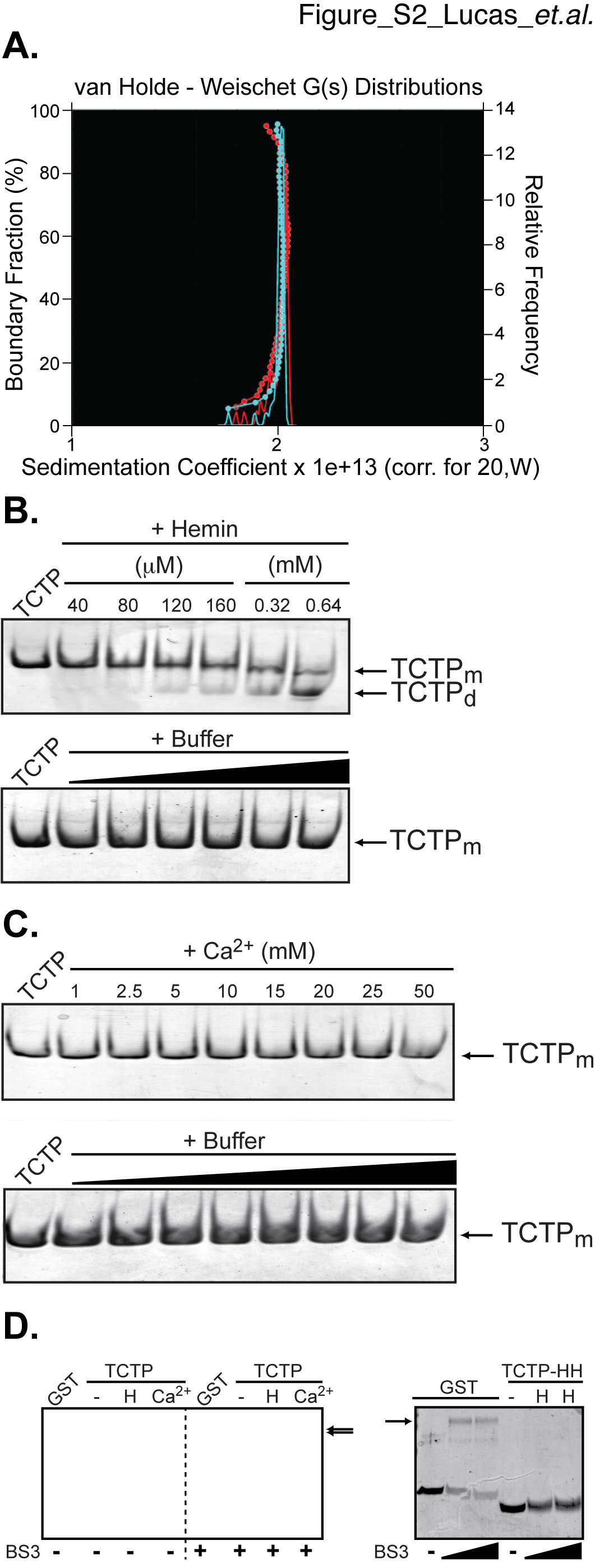

Supplement: Figure S2 — A. van Holde – Weischet G(s) distribution of TCTP species at two different concentrations (ODs 045 and 1.3). B–C. Electrophoretic profile of TCTP separated by native gel electrophoresis and visualized by Coomassie blue staining. In all cases, TCTP (8 µg) was pre-incubated with various concentrations of either hemin (B; 40 to 640 µM) or Ca2+ (C; 1 to 50 mM) before being loaded onto the gel. In each scenario, buffers were used as controls. D. Chemical crosslinking of GST (positive control), TCTP (left panel), or TCTP-HH (right panel; 5 µg each) and either hemin (H; 32 µM) or CaCl2 (Ca2+; 50 mM) in the presence (+) or absence (-) of BS3 as described in “Materials and Methods”. Right panel: GST and TCTP-HH were tested with two different concentrations of BS3 (1 and 3 mM) and samples were resolved by SDS-PAGE. Arrows indicate dimeric complexes. (TIF) [file pone.0112823.s002.tif]

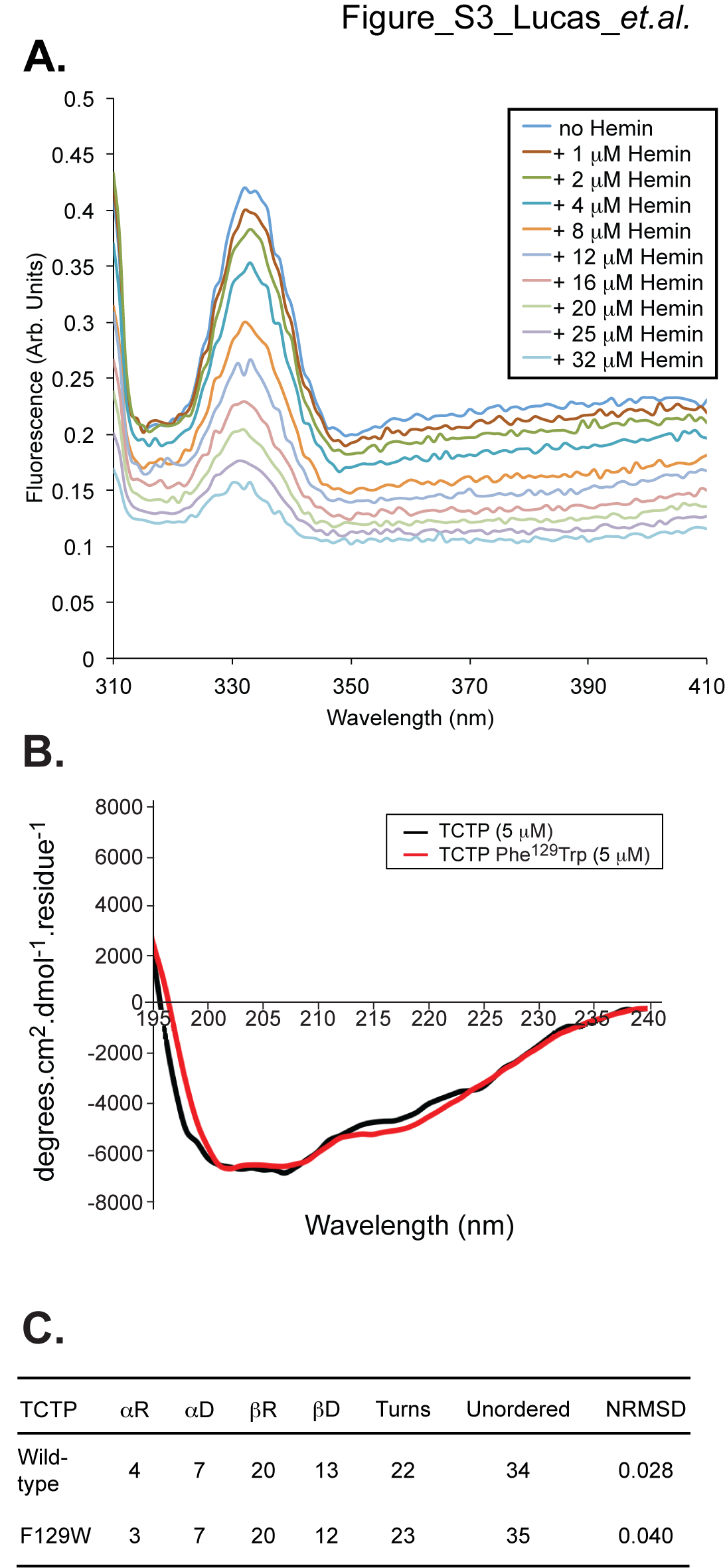

Supplement: Figure S3 — A. Background spectra for the various concentrations of hemin (1–32 µM) tested in Figure 2D. B. Far-UV circular dichroism spectra of TCTP (5 µM, black line) and TCTP Phe129Trp mutant (5 µM, red line) at pH 6.8, 298°K. C. Predicted secondary structure content of TCTP and TCTP Phe129Trp mutant using the CDSSTR algorithm. R and D represent regular and distorted secondary structure elements, respectively. NRMSD: normalized root mean square. (TIF) [file pone.0112823.s003.tif]

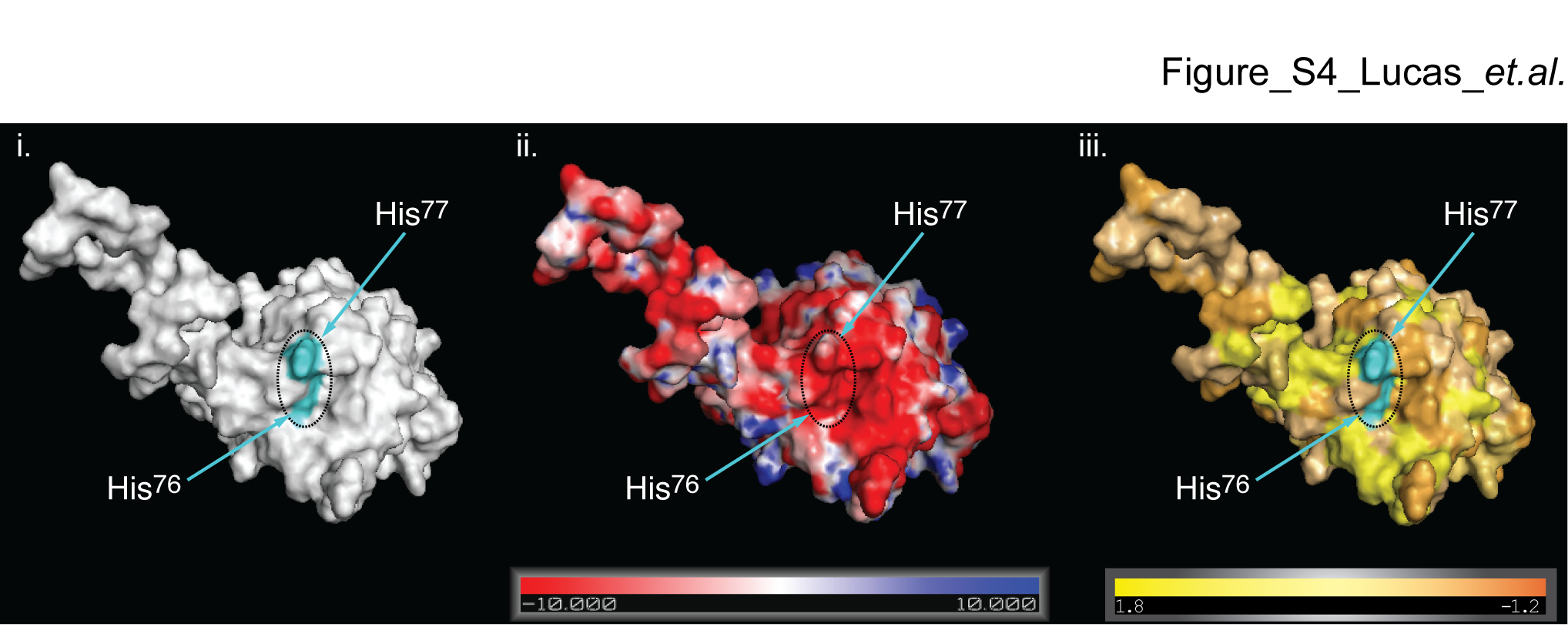

Supplement: Figure S4 — Surface representation of TCTP (PDB access code: 1YZ1) depicting the position of residues His76 and His77 (in cyan, panel i ) and colored according to electrostatic charges (negative and positive potentials in red and blue, respectively; panel ii ) and conservation scores based on interface propensity to bind heme shown in panel iii [47] . (TIF) [file pone.0112823.s004.tif]

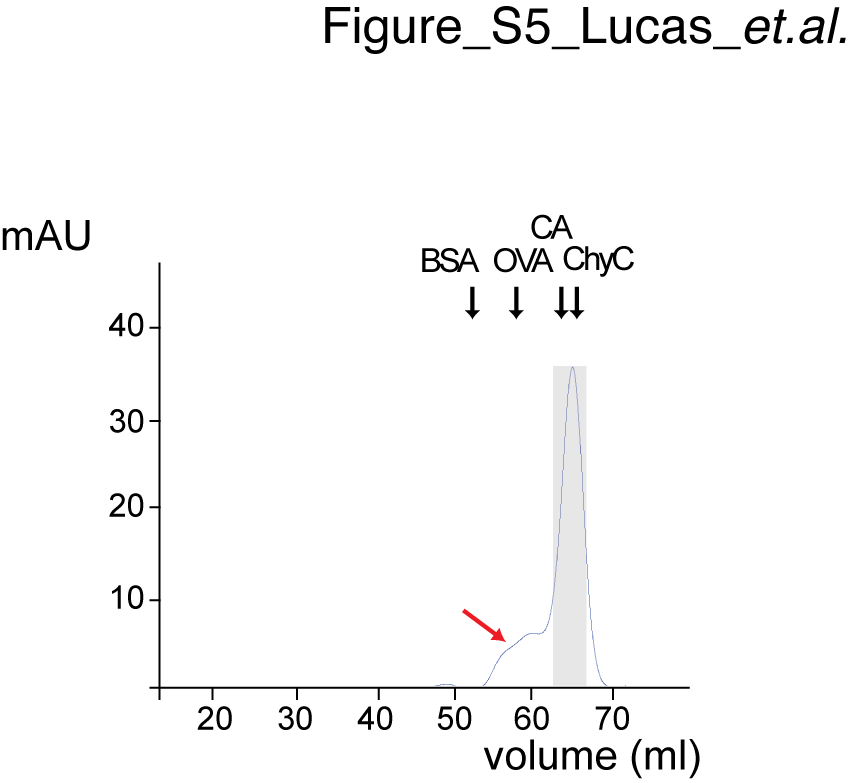

Supplement: Figure S5 — Elution profile of recombinant untagged-TCTP pooled from fractions 65–68 from Figure 1A and resolved by gel filtration using a 16/60 Superdex 75 column pre-equilibrated with 50 mM CaCl2. Red arrow indicates additional oligomeric forms present in the sample. (TIF) [file pone.0112823.s005.tif]
