# Supplementary material for: Ligand Binding Reveals a Role for Heme in Translationally-Controlled Tumor Protein Dimerization
Source: PLoS One. 2014 Nov 14;9(11):e112823. doi: 10.1371/journal.pone.0112823 (PMC4232476; doi:10.1371/journal.pone.0112823)
Supplement: Table S1 — Summary of heme-protected TCTP fragments detected by MS/MS. Results were obtained by digestion with individual proteases followed by MS/MS analysis. Protease are indicated with a one letter code and are: G: endoproteinase GluC; L: endoproteinase LysC; C: Chymotrypsin; T: Trypsin. (DOCX) [file pone.0112823.s006.docx]

**Table S1. *Summary of heme-protected TCTP fragments detected by MS/MS.***

| **Peptide Range** | **Peptide Sequence** | **Modif.** | **Observed**  **m/z** | **Mass error (ppm)** | **Protease** | **Peptide termini** | **Confirmed by MSMS** |
| --- | --- | --- | --- | --- | --- | --- | --- |
| 40-81 | R.TEGNIDDSLIGGNASAEGPEGEGTESTVITGVDIVMNHHLQE.T |  | 4293.1885 | 50 | G | T-G | N |
| 40-81 | R.TEGNIDDSLIGGNASAEGPEGEGTESTVITGVDIVMNHHLQE.T | ox Met | 4309.1040 | 31 | G | T-G | N |
| 49-84 | L.IGGNASAEGPEGEGTESTVITGVDIVMNHHLQETSF.T |  | 3683.8115 | 27 | C | C-C | N |
| 49-84 | L.IGGNASAEGPEGEGTESTVITGVDIVMNHHLQETSF.T | ox Met | 3699.7625 | 15 | C | C-C | N |
| 65-81 | E.STVITGVDIVMNHHLQE.T |  | 1893.0051 | 27 | G | G-G | Y |
| 65-81 | E.STVITGVDIVMNHHLQE.T | ox Met | 1908.9910 | 22 | G | G-G | Y |
| 76-84 | M.NHHLQETSF.T |  | 1112.5238 | 11 | C | C-C | Y |
| 92-98 | K.YIKDYMK.S |  | 960.5012 | 16 | L | L-L | Y |
| 92-98 | K.YIKDYMK.S | ox Met | 976.4970 | 17 | L | L-L | Y |
| 99-111 | K.SIKGKLEEQRPER.V |  | 1569.8942 | 15 | L | L-T | Y |
| 102-111 | K.GKLEEQRPER.V |  | 1241.6935 | 27 | L | L-T | Y |
| 104-111 | K.LEEQRPER.V |  | 1056.5741 | 29 | T | T-T | Y |
